# Detection of fossil-fuel CO_2_ plummet in China due to COVID-19 by observation at Hateruma

**DOI:** 10.1038/s41598-020-75763-6

**Published:** 2020-10-29

**Authors:** Yasunori Tohjima, Prabir K. Patra, Yosuke Niwa, Hitoshi Mukai, Motoki Sasakawa, Toshinobu Machida

**Affiliations:** 1 National Institute for Environmental Studies, 16-2 Onogawa, Tsukuba, Ibaraki 305-8506 Japan; 2 Japan Agency for Marine-Earth Science and Technology (JAMSTEC), 3173-25 Syowa-machi, Kanazawa-ku, Yokohama, 236-0001 Japan

**Keywords:** Climate sciences, Environmental sciences, Environmental social sciences

## Abstract

The COVID-19 pandemic caused drastic reductions in carbon dioxide (CO_2_) emissions, but due to its large atmospheric reservoir and long lifetime, no detectable signal has been observed in the atmospheric CO_2_ growth rate. Using the variabilities in CO_2_ (ΔCO_2_) and methane (ΔCH_4_) observed at Hateruma Island, Japan during 1997–2020, we show a traceable CO_2_ emission reduction in China during February–March 2020. The monitoring station at Hateruma Island observes the outflow of Chinese emissions during winter and spring. A systematic increase in the ΔCO_2_/ΔCH_4_ ratio, governed by synoptic wind variability, well corroborated the increase in China’s fossil-fuel CO_2_ (FFCO_2_) emissions during 1997–2019. However, the ΔCO_2_/ΔCH_4_ ratios showed significant decreases of 29 ± 11 and 16 ± 11 mol mol^−1^ in February and March 2020, respectively, relative to the 2011–2019 average of 131 ± 11 mol mol^−1^. By projecting these observed ΔCO_2_/ΔCH_4_ ratios on transport model simulations, we estimated reductions of 32 ± 12% and 19 ± 15% in the FFCO_2_ emissions in China for February and March 2020, respectively, compared to the expected emissions. Our data are consistent with the abrupt decrease in the economic activity in February, a slight recovery in March, and return to normal in April, which was calculated based on the COVID-19 lockdowns and mobility restriction datasets.

## Introduction

The outbreak of the new coronavirus (COVID-19) was first identified in Wuhan, China, in December 2019. The government of China took a range of measures including a lockdown of Wuhan, shut-down of the inter-city transportation, and reduction of socioeconomic activity at the end of January 2020 to prevent the spread of COVID-19 within China and to the outside world. As a result of these measures, it was estimated that the emissions of fossil-fuel-derived CO_2_ (FFCO_2_) in China decreased by about 25% during January–February^1,2^. Significant reductions in nitrogen dioxide (NO_2_), which is an atmospheric pollutant mainly produced by fossil fuel combustion in engines, were also detected over China by remote sensing satellites^3,4^. However, no observational evidence has been reported about the detection of China’s emission reduction in atmospheric CO_2_ concentrations. It is also unclear whether atmospheric observations can support the direct estimation of reductions in FFCO_2_ emissions in near-real-time.

A global observation network for atmospheric greenhouse gases has been developed since the 1960s^5^ not only to evaluate the global anthropogenic and natural flux budgets but also to quantitatively estimate the regional/country-scale emission changes^6–9^. Therefore, detecting the COVID-19 influence on atmospheric CO_2_ from observations and, if possible, quantitatively evaluating the emission change in China is crucially important to test the capacity of our observation networks. National restrictions owing to the COVID-19 situation give us a unique opportunity to validate some of the hypotheses that are needed to successfully implement the Paris Agreement.

The National Institute for Environmental Studies (NIES)/Center for Global Environment Research (CGER) has been conducting global monitoring of atmospheric greenhouse gases by using a variety of platforms, including ground sites^10^, commercial cargo ships^11,12^, aircraft^13,14^, and satellites^15,16^. As a part of the global monitoring effort, NIES/CGER operates an observation station at Hateruma Island (HAT), Japan, to carry out comprehensive atmospheric measurements. Since the island is located in a marginal region of continental East Asia, the outflow of the continental air masses with elevated greenhouse gas concentrations is often captured at HAT. Previous studies revealed that the synoptic-scale variations during the winter season can be used to constrain the emissions from continental East Asia^17^. Here, we examine whether the reduced economic activity in China has caused any detectable change in the CO_2_ synoptic variations at HAT. Synoptic variations are defined as the hourly to weekly variations in the CO_2_ and CH_4_ time series, and their variabilities are termed as ΔCO_2_ and ΔCH_4_, respectively (see “Methods” section).

## Results

### Anomalous behavior of CO_2_ in February 2020

**Figure 1:**
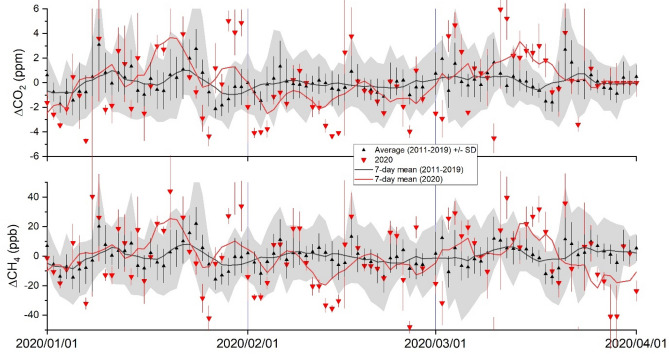
Variabilities in atmospheric CO_2_ and CH_4_ mole fractions observed at HAT. The detrended and deseasonalized CO_2_ (top) and CH_4_ (bottom) mole fractions observed at HAT from January to March 2020 are compared with the corresponding 9-year averages. The red triangles and red bars are the daily means and the standard deviation (1-σ) for 2020, the black triangles and black bars are the 9-year average of the daily mean and daily standard deviation for the corresponding day of the year, and the grey shade is the standard deviation (1-σ) of the 9-year data. The red and black solid lines represent the 7-day moving averages of the daily means in 2020 and the 9-year averages, respectively.

In Fig. 1, daily means of the detrended and deseasonalized atmospheric CO_2_ and CH_4_ mole fractions (see “Methods” section) observed at HAT in January-March 2020 are compared with those of the previous 9-year (2011–2019) average. Closely investigating these data, we found that February 2020 was the only occasion when CO_2_ was systematically lower than the long-term (2011–2019) mean for 22 days out of the 29 days of the month, and 9 days in the 2020 values fell outside the 1-σ standard deviation range of the 2011–2019 average. A large fraction of the variabilities in CO_2_ (ΔCO_2_) at HAT are affected commonly by the movement of emission signals from China by air mass transport, which can be analyzed effectively by air mass trajectories (Fig. S1 in Supplementary Material). Due to the common high emissions of CO_2_ and CH_4_ over China and air mass trajectories between China and HAT, similar variabilities were observed also for CH_4_ (ΔCH_4_) in February 2020, although the frequency of lower value cases was fewer than those for CO_2_. Such anomalies were emphasized when we compared the 7-day moving averages between the daily means and the 9-year averages (Fig. 1).

However, it is quite difficult to relate such anomalies to the change in FFCO_2_ emissions. The atmospheric CO_2_ signal due to changes in FFCO_2_ weakens by atmospheric transport as the distance between the source and observation site (or receptor), i.e., HAT, increases. Yet the rather simple air mass trajectories and the suppressed terrestrial biospheric exchange in the winter months allow us to evaluate the FFCO_2_ sources in China from the synoptic-scale variabilities at HAT^17,18^. In the rest of the analysis, we discuss the ratios of ΔCO_2_ and ΔCH_4_ for removing the first-order effect of atmospheric transport on the CO_2_ synoptic variability. This study was based on the assumption that the CO_2_ and CH_4_ flux signals had similar spatial distributions in the outflow region of China during the winter through spring, and thus produced coinciding peaks and troughs in atmospheric variability observed at HAT^17,18^.

### 25-year change in the ΔCO_2_/ΔCH_4_ ratio in winter

**Figure 2:**
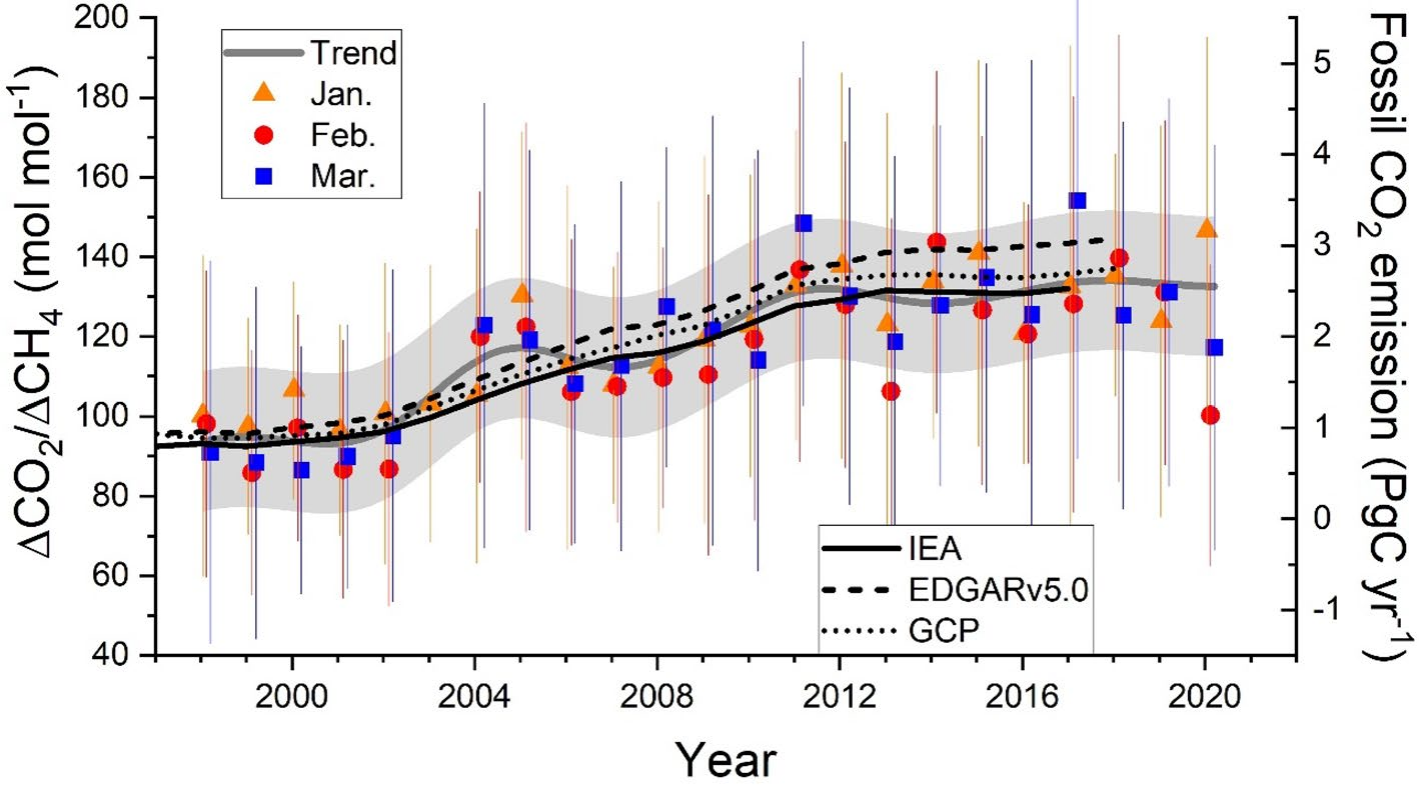
Temporal variations in the monthly average ΔCO_2_/ΔCH_4_ ratios for January, February, and March since 1996. The grey thick line represents the trend curve of the ΔCO_2_/ΔCH_4_ ratio based on a digital filtering technique^25^ with a cut-off period of five years and the grey-shaded area represents the 95% range of the variations from the trend curve. Thin lines are the estimation of FFCO_2_ emissions from China based on IEA, EDGARv5.0, and GCP. The position of the right y-axis is adjusted so that the FFCO_2_ temporal variations visually fit the trend curve of the ΔCO_2_/ΔCH_4_ ratio. The vertical bars represent the standard deviations (1σ) for the monthly values.

The temporal variations in the monthly average of the ΔCO_2_/ΔCH_4_ ratio for January, February, and March after 1998 are shown in Fig. 2. The average ΔCO_2_/ΔCH_4_ ratios for the three months show an increasing trend during 2001–2011 and reach a plateau after 2011. An analysis using the Lagrangian particle dispersion model (LPDM) revealed that emissions from the northeastern and eastern parts of China predominantly contributed to the increase of the ΔCO_2_/ΔCH_4_ ratio at HAT when averaged over November through March^17,18^. For comparison, we have also plotted the FFCO_2_ inventory emission estimates for China using data from the International Energy Agency (IEA)^19^, Emission Database for Global Atmospheric Research (EDGARv5.0)^20^, and Global Carbon Project (GCP)^21^. The trend of the observed ΔCO_2_/ΔCH_4_ ratio is similar to the increasing trend of the FFCO_2_ estimates. Therefore, the rapid increase in the ΔCO_2_/ΔCH_4_ ratio during the 2000s has been attributed to the rapid increase in fossil fuel consumption associated with the unprecedented economic growth in China. Although a steady increase in anthropogenic CH_4_ emissions was estimated from China during the 2000s^22–24^, the increasing trend of ΔCO_2_/ΔCH_4_ indicates that the relative growth rate of the CO_2_ emissions has exceeded that of the CH_4_ emissions. The rather stable ΔCO_2_/ΔCH_4_ ratio after 2011 suggests a consistent and slower increase of both FFCO_2_ and CH_4_ emissions from China (Fig. 2). Thus, we used the period of 2011–2019 as the reference period for analyzing the 2020 emission change.

The ΔCO_2_/ΔCH_4_ ratio for February 2020 shows a significant decrease beyond the 95% confidence interval. The histograms of the individual ΔCO_2_/ΔCH_4_ ratios of the 24-h time windows for February show that the histogram for 2020 shifts slightly toward a smaller ΔCO_2_/ΔCH_4_ ratio compared to the recent decade (2011–2019) (Fig. S2 in Supplementary Material). The ΔCO_2_/ΔCH_4_ ratio of February 2013 also shows a significant decrease. Investigating the back trajectories arriving at HAT in February during the recent decade (Fig. S1 in Supplementary Material), we found that air masses were occasionally transported from Southeast Asia in February 2013, a region with lower CO_2_/CH_4_ molar emission ratios than the East Asian countries. Thus, the low ΔCO_2_/ΔCH_4_ ratio in February 2013 could be attributed to irregular air mass transport. Since such an irregularity was not confirmed in February 2020, we consider that a reduction in the FFCO_2_ emissions from China caused the observed decrease.

### Temporal variation in the ΔCO_2_/ΔCH_4_ ratio from December 2019 through April 2020

**Figure 3:**
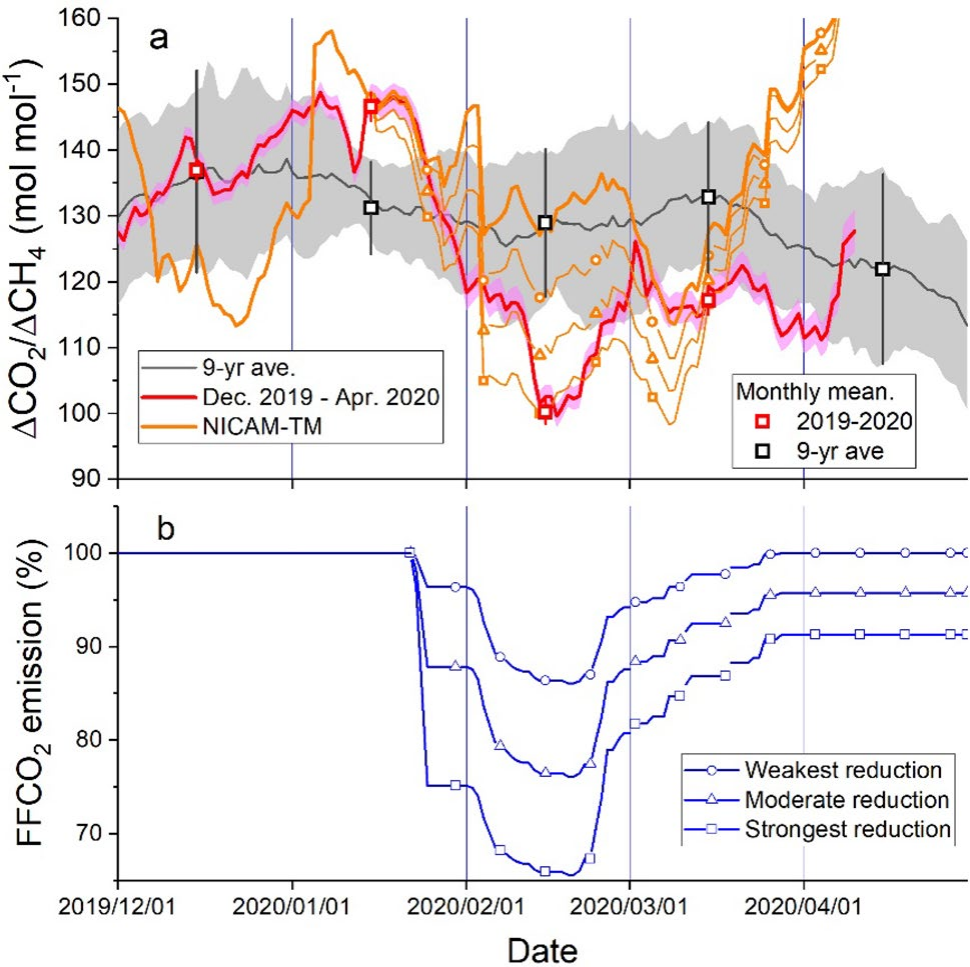
Temporal variations in ΔCO_2_/ΔCH_4_ ratios and estimated FFCO_2_ emissions. (**a**) The red line represents the 30-day moving average of the ΔCO_2_/ΔCH_4_ ratios from December 2019 to April 2020, the black line with grey shade represents the 9-year (2011–2019) average of the 30-day moving average and the range of the variation (± 1σ), and the orange lines represent the results of the NICAM-TM simulation, corresponding to the control emission case (no symbols) in comparison with the weakest (circles), moderate (triangles) and strongest (squares) FFCO_2_ reduction cases. The red and black open squares represent the monthly means of the ΔCO_2_/ΔCH_4_ ratios and the 9-year (2011–2019) average of the monthly means. (**b**) The blue lines represent the temporal variations of estimated FFCO_2_ emission change in China based on the restriction levels for the 30 Chinese provinces given by Le Quéré et al.^2^.

The temporal changes in the 30-day moving-window average of ΔCO_2_/ΔCH_4_ ratio are shown in Fig. 3. The 9-year (2011–2019) average ratios show a slight decline from January to February. As the period between late January and early February corresponds to the Chinese New Year, the decline might be attributed to the decrease in FFCO_2_ emissions from China associated with the seasonal decrease in economic activity. The 9-year average also shows a decreasing trend in April, due mainly to a gradual increase in biogenic CH_4_ emissions (Fig. S3 in Supplementary Material). Although the 30-day-averaged standard deviations for CO_2_ and CH_4_ (ΔCO_2_ and ΔCH_4_) show basically similar patterns of temporal variations (Fig. S3 in Supplementary Material), the 30-day-averaged ΔCO_2_/ΔCH_4_ ratios show a sharp decrease from January to February 2020. The January anomaly exceeds the range of the 9-year average, reaches a minimum in mid-February, and then gradually moves toward the 9-year average in March. The observed results suggest a rapid recovery in the FFCO_2_ emissions from China by the end of March, which are consistent with recent FFCO_2_ emission estimates based on the activity data from power generation and industry (https://carbonmonitor.org/).

We examined whether the observed change in the ΔCO_2_/ΔCH_4_ ratio reflected the FFCO_2_ emission change in China on a day-to-month time scale with respect to the pre-pandemic emission level (Fig. 3). The temporal change in the relative FFCO_2_ emissions in China is shown in Fig. 3, which was calculated from the time series of the levels of measures against the virus for the Chinese provinces given by Le Quere et al.^2^ (see “Methods” section). The observed ΔCO_2_/ΔCH_4_ ratio traces amazingly well the change in the relative FFCO_2_ emissions from China. This result validates convincingly the hypothesis that the ΔCO_2_/ΔCH_4_ ratio at HAT can track changes in FFCO_2_ emissions from China on a shorter time scale during the winter months when the terrestrial biosphere is in hibernation.

To further investigate whether the estimated FFCO_2_ change could explain the observed changes in the ΔCO_2_/ΔCH_4_ ratio, an atmospheric transport model named NICAM-TM^26^ was used to estimate the changes in the ratios (see “Methods” section). Globally distributed CO_2_ fluxes included emissions from fossil-fuel combustion and cement production, terrestrial biospheric exchange and air-sea exchange, and CH_4_ fluxes comprised of both anthropogenic and natural emissions. In addition to the control simulation, three sensitivity simulations were performed with modified FFCO_2_ emissions in China, in which the emissions were reduced to 70%, 80%, and 89% following the lock-down intensity time series at daily intervals with the strongest, modest, and weakest assumptions, respectively. In March, these reductions were relaxed to 87%, 92%, and 98%, respectively. The temporal changes in the ΔCO_2_/ΔCH_4_ ratios, which were computed from the simulated data at an hourly time interval, are also shown in Fig. 3. Although being more variable than the observations, the differences in the simulated ratios for the moderate and strongest reduction cases from that of the control case reproduced well the rapid decrease between January and February 2020. Note that the rather large variability in the simulated ΔCO_2_/ΔCH_4_ ratio is in part attributed to the relatively small variability in the simulated CH_4_. Among the simulation results of the three FFCO_2_ reduction cases, which correspond to the bottom, middle, and top lines of the FFCO_2_ emission estimates shown in Fig. 3, the strongest reduction (70% reduction) case explains best the observed reduction in February 2020.

In the NICAM-TM simulations, we used climatological CO_2_ fluxes from the terrestrial biosphere to calculate the ΔCO_2_/ΔCH_4_ ratios (see “Methods” section). Thus, the temperature-dependent interannual variability in heterotrophic respiration was ignored in the model simulation. An analysis of the surface air temperature anomaly did not show any large change over the East China region in February–March 2020 (Fig. S4 in Supplementary Material). The simulated monthly ΔCO_2_/ΔCH_4_ ratios for February and March based on the time-dependent FFCO_2_ emissions and the climatological biospheric CO_2_ fluxes generally well reproduced the observed temporal variations over the period of 2000–2019 (see Fig. S5 in Supplementary Material). From these facts, we conclude that the observed decrease in the ΔCO_2_/ΔCH_4_ ratios at Hateruma was caused predominantly by the change in FFCO_2_ emissions rather than the change in the terrestrial biosphere.

### Estimation of monthly FFCO_2_ emissions from China

**Figure 4:**
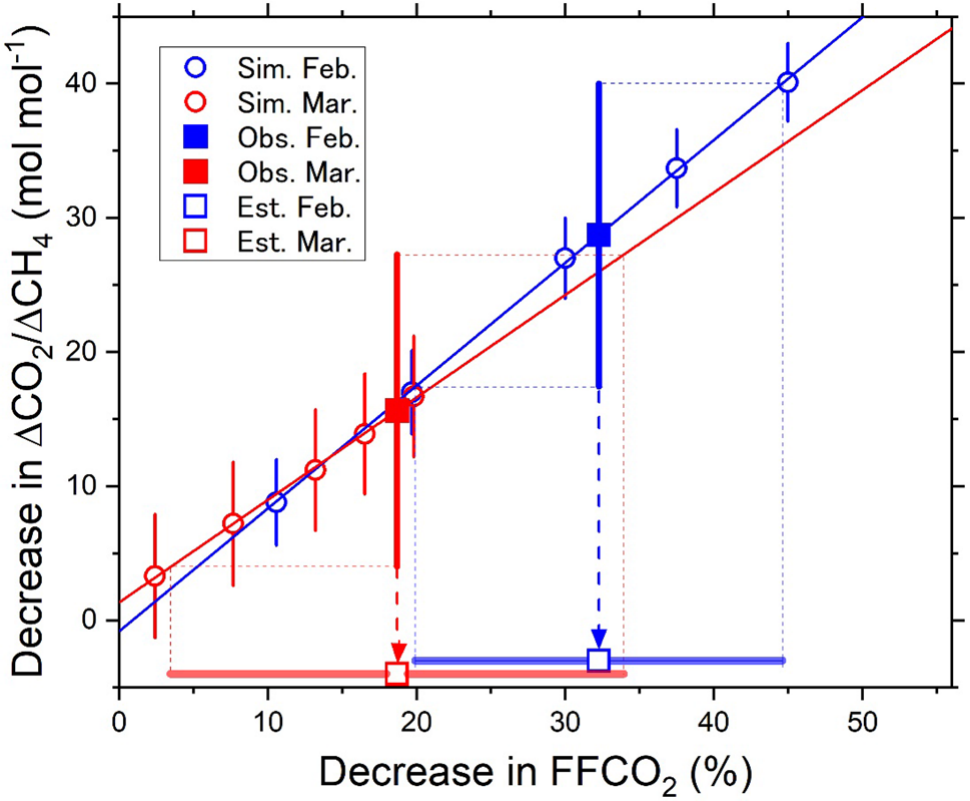
Estimation of the decrease in FFCO_2_ emissions in China. The relationship between the relative decrease in FFCO_2_ emissions from China and the decrease in the simulated ΔCO_2_/ΔCH_4_ ratios is plotted as open circles (blue: February; red: March). The blue and red lines represent the linear regression lines of the simulated data in February and March, respectively. The decreases in the observed ΔCO_2_/ΔCH_4_ ratios are applied to the linear regression lines to obtain the estimates for the FFCO_2_ decrease.

We estimated the FFCO_2_ emission decreases caused by the influence of the COVID-19 outbreak in China in February and March by using the observed differences between the monthly-mean ΔCO_2_/ΔCH_4_ ratios in 2020 and the 9-year (2011–2019) averages (Fig. 3, Table 1). The observed decreases in the ΔCO_2_/ΔCH_4_ ratios were 29 ± 11 mol mol^−1^ (from 129 ± 11 to 100 ± 2 mol mol^−1^) in February 2020 and 16 ± 11 mol mol^−1^ (from 133 ± 11 to 117 mol mol^−1^) in March 2020. The influence of the FFCO_2_ reductions on the ΔCO_2_/ΔCH_4_ ratio can be evaluated using NICAM-TM simulations as well (see “Methods” section and Table S1 in Supplementary Material). Applying the observed decreases to the linear relationships between the decreases in the FFCO_2_ emissions and the simulated changes in the ΔCO_2_/ΔCH_4_ ratio for the individual months, we obtained the estimates of the relative FFCO_2_ emission decrease of 32 ± 12% in February and 19 ± 15% in March (Fig. 4, Table 1). These estimates are close to the upper limits for the reduction of the activity-based estimations^2^, which are 20% (11 to 30%) in February and 8% (2 to 13%) in March.

**Table 1:** Change in the observed ΔCO_2_/ΔCH_4_ ratio and estimated FFCO_2_ decrease.

Date (year/month)	Monthly average of ΔCO_2_/ΔCH_4_ (mol mol^−1^)	9-year average of ΔCO_2_/ΔCH_4_ (mol mol^−1^)	Estimated decrease in FFCO_2_ (%) (This study)	Estimated FFCO_2_ decrease based on Le Quere et al.^2^ (%)
2020/02	100 ± 2	129 ± 11	32 ± 12	20 (11–30)
2020/03	117 ± 2	133 ± 11	19 ± 15	8 (2–13)

In the calculations, we assumed that the regional pattern of the distribution of FFCO_2_ emissions was stable, therefore, we used a single scaling factor to adjust the FFCO_2_ emissions from China. However, regional variations in the distribution of the FFCO_2_ emissions could also affect the ΔCO_2_/ΔCH_4_ ratios at HAT as was discussed in detail in a previous study^17^, which showed that different FFCO_2_ emission maps caused significant differences in the ΔCH_4_/ΔCO_2_ ratios. Furthermore, as was described in the previous section, the observation at HAT is not sensitive to the FFCO_2_ emissions from central and western China. Therefore, it should be noted that these limitations would introduce additional errors in the estimated FFCO_2_ emissions, which are discounted in this study.

For the NICAM-TM simulations, we have assumed that CH_4_ emissions from China have not changed due to the COVID-19-related economic slowdown. However, there is a possibility that among the major sources including coal exploitation (32%), wastewater handling (13%), enteric fermentation (13%), oil and natural gas exploitation (5%), and solid waste disposal (5%), CH_4_ emissions from fossil fuel exploitation were also reduced following the reduction in fossil fuel consumption. In a sensitivity simulation, the fossil-fuel-related CH_4_ emissions, which account for about 37% of the total CH_4_ emissions from China in winter, were reduced at the same rate as the FFCO_2_ reduction due to the COVID-19 influence. Such a reduction in CH_4_ emissions corresponding to the moderate case of FFCO_2_ reduction in China resulted in increases in the ΔCO_2_/ΔCH_4_ ratios in February and March by 28% and 26%, respectively. Taking into account these results, the decrease in the observed ΔCO_2_/ΔCH_4_ ratios may suggest that either the decrease in the CH_4_ emissions was not large or the fossil-fuel-derived CO_2_ reduction was much larger than the estimations. Nevertheless, we consider that the former is a more plausible explanation. This is because (1) FFCO_2_ emissions are not directly associated with the above-mentioned anthropogenic CH_4_ emissions and (2) it is difficult to rapidly (within a few months) change the CH_4_ seepage from inundated coal mines as well as the biogenic CH_4_ emissions from wastewater, solid waste and enteric fermentation.

Recently, the UN environment programme discussed the effect of COVID-19 on the growth rate of the global CO_2_ concentrations^27^. It is quite difficult to forecast the annual decrease in the global FFCO_2_ emissions in 2020 because it depends on a variety of factors: the duration of the COVID-19 outbreak, the extent of the restrictions on socioeconomic activity, and the recovery track of the economic activity after the outbreak. A recent estimate based on the plausible range of possibilities shows that the decrease in estimated annual emissions in 2020 ranges from − 5.3 to − 7.5%. Considering the present level of the global FFCO_2_ emissions (about 10 PgC year^−1^), even a 10% reduction of the global emissions results in an atmospheric decrease of only about − 0.5 ppm. Given that CO_2_ increases by about 2 ppm per year with a large interannual variability (up to 70%)^28^ due to the biosphere-climate feedbacks, we will not be able to distinguish easily the effect of FFCO_2_ emission reduction on the atmospheric CO_2_ growth rate in 2020 without a very highly accurate estimation of the land and oceanic uptakes. Our model sensitivity simulation using the control and the largest reduction scenario (**− **30% FFCO_2_ emissions in February) cases suggested a maximum of 0.8 ppm in the total column CO_2_ (XCO_2_) change over China and the signal from emission reductions spread to southwest and northeast directions by the atmospheric transport (Fig. S6 in Supplementary Material). Given the small difference of XCO_2_, the existing remote sensing satellites, the GOSAT and OCO series with single-shot precision of about 2 ppm^16,29^, will face challenges in detecting any COVID-19 effect. However, our method can detect large signals from the emission reduction from any specific region (China) in near-real-time using continuous, simultaneous, and high-precision measurements of CO_2_ and CH_4_.

## Methods

### Atmospheric observation at HAT

Continuous monitoring of atmospheric CO_2_ and CH_4_ has been carried out at HAT since 1993 and 1996, respectively. In this study, the sample air was drawn from the top of a tower at the height of 36.5 m (46.5 m above sea level), dried by passing through dehumidifiers, and then introduced into the individual measurement systems. Atmospheric CO_2_ was continuously measured by using a nondispersive infrared spectroscopic analyzer (NDIR), whereas atmospheric CH_4_ was semi-continuously measured by a gas chromatograph (GC) equipped with a flame ionization detector (FID). These measurement systems were calibrated against several standard gases supplied from high-pressure cylinders, and CO_2_ and CH_4_ mole fractions were carefully determined against NIES’s original mole fraction scales^11,30^. The precisions of CO_2_ and CH_4_ were ~ 0.1 μmol mol^−1^ (ppm) and ~ 2 nmol mol^−1^ (ppb), respectively. The details of the measurements have been reported elsewhere^9,10^. Note that the analytical interval of the GC/FID system before December 1997 was 1.5 times longer than that after December 1997. Thus, the data after December 1997 were used in this study as was done in a previous study^17^.

Since 2013, a cavity ring-down spectroscopic analyzer (CRDS, Picarro G-2401) has also been deployed at the monitoring station at HAT to back up the atmospheric observation of CO_2_, CH_4,_ and CO. The CO_2_ mole fractions determined by CRDS and NDIR show considerable compatibility; the mean and the standard deviation of the difference in the hourly CO_2_ mole fractions (NDIR–CRDS) for the period of January 2019–March 2020 was 0.01 ± 0.3 ppm. Since the NDIR system was broken during December 19–30, 2019, and March 4–24, 2020, the data by CRDS were used to fill the data gaps in this study.

An example of the observed synoptic variations of atmospheric CO_2_ and CH_4_ at HAT is shown in Fig. S7 in Supplementary Material, where the detrended and deseasonalized time series computed based on a digital filtering technique^25^ between January and March 2018, 2019, and 2020 are depicted. Note that the right y-axis for CH_4_ is upside down. It should also be noted that the mean CO_2_ variations at HAT didn’t show a significant diurnal cycle because of the relatively small influences from local sources or local meteorology during winter as well as CH_4_^10^. The figure clearly shows that there exists an excellent similarity in both temporal variations. During winter, air masses arriving at HAT are frequently transported from continental East Asia due to the winter East Asian Monsoon. Additionally, there is a rough similarity in the source distributions of the FFCO_2_ and CH_4_ in the East Asian region (e.g. Tohjima et al.^18^). These conditions resulted in excellent correlative synoptic-scale variations in the atmospheric CO_2_ and CH_4_ at HAT.

### Methods of ΔCO_2_/ΔCH_4_ ratio analysis

We didn’t use either baselines or smooth-curve fits to the time series of the atmospheric CO_2_ and CH_4_ to examine synoptic-scale variations because we had difficulty in determining appropriate ones^31^. Instead, we investigated the relative variation ratio by adopting an approach taken by Tohjima et al.^17^. First, we calculated the standard deviations (ΔCO_2_ and ΔCH_4_) and correlation coefficient (R) between CO_2_ and CH_4_ for the data within a 24-h time window. Then, we obtained the ΔCO_2_/ΔCH_4_ ratio from the individual standard deviations. Note that the ΔCO_2_/ΔCH_4_ ratio corresponds to the linear regression slope of the CO_2_ and CH_4_ scatter plot based on the Reduced Major Axis method (RMA)^32^, which takes into account errors in both the independent (x-axis) and dependent (y-axis) variables. This procedure was repeated for the entire data set by shifting the 24-h time window by one hour. Then, the ΔCO_2_/ΔCH_4_ ratios with corresponding correlation coefficients larger than 0.7 (R > 0.7) were used to compute the monthly averages or 30-day moving averages shown in Figs. 2, 3, and 4.

To investigate the individual contributions of ΔCO_2_ and ΔCH_4_ to the temporal variations in the 9-year-averaged ΔCO_2_/ΔCH_4_ ratio shown in Fig. 3, the temporal variations of the 9-year-averaged 30-day moving averages of the ΔCO_2_ and ΔCH_4_ were examined (Fig. S3 in Supplementary Material). In general, ΔCO_2_ and ΔCH_4_ show similar temporal variations, and their slight differences caused the temporal variation in the ΔCO_2_/ΔCH_4_ ratio. The gradually decreasing trend in the ΔCO_2_/ΔCH_4_ ratio after March may be attributed to the larger increase in ΔCH_4_. The 30-day moving averages of the ΔCO_2_ and ΔCH_4_ from December 2019 to April 2020 are also depicted in Fig. S3 in Supplementary Material. Again, there is a considerable similarity between ΔCO_2_ and ΔCH_4_, suggesting that the atmospheric mixing predominantly caused the temporal variability in those atmospheric components.

In this study, we used the value of 0.7 as the criteria of the correlation coefficient (R) and 24 h for the time window (TW) of the correlation analysis. The results shown in this study are slightly influenced by the selection of these values. The estimated FFCO_2_ emissions from China based on the observed monthly mean ΔCO_2_/ΔCH_4_ ratio for the ranges of 0.5 < R < 0.8 and 12 < TW < 48 (hour) are plotted in Fig. S8 in Supplementary Material. On average, there is a slight tendency that the estimated monthly FFCO_2_ emissions increase with increasing values of R and TW. However, the standard deviations of the estimated values for the individual months, being less than 6%, are smaller than the uncertainties associated with the individual estimations (Table 1). Therefore, we ignored the effect of the selection of these values in this study.

### Estimation of the change in daily FFCO_2_ emissions from China based on activity data

The influence of measures against COVID-19 on China’s FFCO_2_ emissions was evaluated based on the approach taken by Le Quere et al.^2^. They categorized the restrictions to the normal economic activity into 4 levels by introducing a confinement index (CI, where CI = 0 is no restriction and CI = 3 is the highest level of restriction). Then, the change in activity for six economic sectors (power, industry, surface transport, public, residential, and aviation) was estimated as a function of the CI level. Note that the mean and the range (lower and upper levels) of the individual activity changes were given for all combinations of the three CI levels and the six economic sectors^2^. We evaluated the change in the FFCO_2_ emissions during January-April 2020 by using the time series of the CI levels for 30 Chinese provinces^2^. As for the FFCO_2_ emissions for individual provinces, we used the data summarized by Shan et al.^33^. We assumed that the proportions of the six economic sectors for the 30 provinces were the same as those for China’s total emissions taken from IEA (2019). The temporal change in the contributions of the individual provinces to the total FFCO_2_ emissions for the moderate case is depicted in Fig. S9 in Supplementary Material. The temporal change in the total FFCO_2_ emissions for the moderate case is also plotted in the figure (right y-axis).

### Simulation of the change in atmospheric CO_2_ and CH_4_ at HAT

Atmospheric CO_2_ and CH_4_ mole fractions at HAT were simulated by using a three-dimensional atmospheric transport model, NICAM-TM^26^, and a comprehensive set of global CO_2_ and CH_4_ fluxes. NICAM-TM was developed from the Nonhydrostatic ICosahedral Atmospheric Model (NICAM)^34^ to examine the atmospheric transport and flux inversion studies of greenhouse gases^35^. The model with a horizontal resolution (mean grid interval) of about 112 km used in this study was driven by nudging horizontal winds towards the data of the Japanese 55-year Reanalysis (JRA-55)^36^. For the FFCO_2_ flux maps, we used the Open-source Data Inventory for Anthropogenic CO_2_ (ODIAC) of version 2019 (ODIAC2019), which is a global high-resolution FFCO_2_ data product^37,38^. We used monthly mean air-sea CO_2_ flux maps prepared by the Japan Meteorological Agency (JMA)^39,40^ and monthly biomass burning CO_2_ flux maps from the Global Fire Emission Database version 4 s (GFED4s, van der Werf et al. 2017)^41^. Note that we used the latest flux maps from ODIAC (2018), JMA (2018), and GFED4s (2016) to simulate the atmospheric CO_2_ mole fractions in 2019 and 2020. In addition, we used averaged monthly biospheric CO_2_ flux maps based on the inversion flux dataset during 2006–2008. As for the CH_4_ flux maps, we used an inversion flux dataset derived from the NICAM-TM 4D-var system^42,43^. These inversion flux maps were prepared to participate in a multi-disciplinary study aimed at global CH_4_ budget estimation under the Global Carbon Project (GCP)^44^. Similarly, the monthly CH_4_ flux maps for 2017 were used to simulate the atmospheric CH_4_ variation in 2020 because of the limitation of the inversion duration. The simulated synoptic-scale variations in CO_2_ and CH_4_ are compared with the observed variations in Fig. S10 in Supplementary Material, where detrended data are plotted.

### Estimation of the relationship between FFCO_2_ emissions and ΔCO_2_/ΔCH_4_ ratio

The relationship between the FFCO_2_ emissions from China and the ΔCO_2_/ΔCH_4_ ratios at HAT was estimated based on the NICAM-TM simulation. In addition to the simulation described in the above section (control case), we conducted three simulations for the three reduction cases (lower, moderate, and upper cases) of the FFCO_2_ emissions from China^2^. Furthermore, to cover the range of the observed ΔCO_2_/ΔCH_4_ changes, we conducted another two simulations for extreme FFCO_2_ reduction cases, in which the FFCO_2_ reduction rates were set to 125% and 150% of those for the upper case. Table S1 in Supplementary Material lists the monthly averages of the decreases in the FFCO_2_ emissions and the corresponding ΔCO_2_/ΔCH_4_ ratios based on the simulations for February and March 2020.

## Supplementary information

Supplementary Information.

## Data Availability

Continuous observations of CO_2_ and CH_4_ time series are available through the NIES database (website). https://db.cger.nies.go.jp/portal/geds/atmosphericAndOceanicMonitoring?lang=eng.
