# Supplementary material for: Detection of fossil-fuel CO2 plummet in China due to COVID-19 by observation at Hateruma
Source: Sci Rep. 2020 Oct 29;10:18688. doi: 10.1038/s41598-020-75763-6 (PMC7596474; doi:10.1038/s41598-020-75763-6)
Supplement: Supplementary file 1 — Supplementary Information. [file 41598_2020_75763_MOESM1_ESM.docx]

Supplementary Materials for

**Detection of fossil-fuel CO_2_ plummet in China due to COVID-19 by observation at Hateruma**

Yasunori Tohjima^1^, Prabir K. Patra^2^, Yosuke Niwa^1^, Hitoshi Mukai^1^, Motoki Sasakawa^1^, Toshinobu Machida^1^

^1^ National Institute for Environmental Studies, 16-2 Onogawa, Tsukuba, Ibaraki 305-8506, Japan

^2^ Japan Agency for Marine-Earth Science and Technology (JAMSTEC), 3173-25 Syowa-machi, Kanazawa-ku, Yokohama 236-0001, Japan

Correspondence to tohjima@nies.go.jp

**Table S1. Simulated change in the ΔCO_2_/ΔCH_4_ ratio as a function of FFCO_2_ change**

| Restriction Case | Feb. 2020 | | Mar. 2020 | |
| --- | --- | --- | --- | --- |
|  | FFCO_2_ reduction | ΔCO_2_/ΔCH_4_ decrease | FFCO_2_ reduction | ΔCO_2_/ΔCH_4_ decrease |
| Lower | 10.6 | 8.8 (3.2) | 2.4 | 3.3 (4.6) |
| Moderate | 19.7 | 17.0 (3.1) | 7.7 | 7.2 (4.6) |
| Upper | 30.0 | 27.0 (3.0) | 13.2 | 11.2 (4.5) |
| 125% of upper | 37.5 | 33.7 (2.9) | 16.5 | 13.9 (4.5) |
| 150% of upper | 45.0 | 40.1 (2.9) | 19.8 | 16.7 (4.5) |

Note: Figures are monthly means and figures in parentheses represent the standard deviations (1σ).


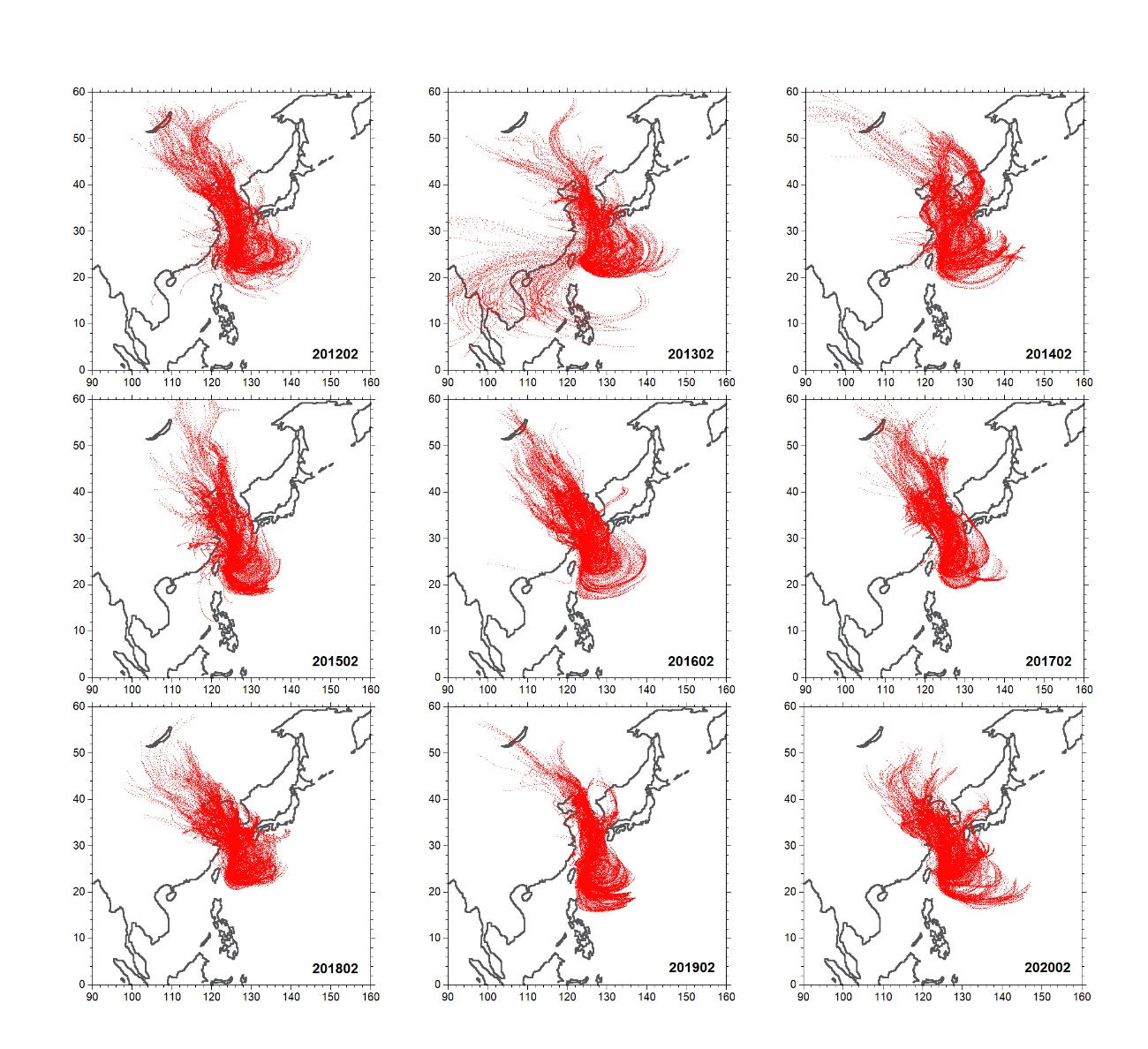


**Fig. S1**. **Air mass arriving at HAT in February of the recent 9 years.** Three-day backward air mass trajectories arriving at HAT in February during 2012-2020. The interval of each trajectory is one hour. The maps were drawn by using Kaleida Graph Ver. 4.5. (Synergy Software, PA, https://www.synergy.com/).


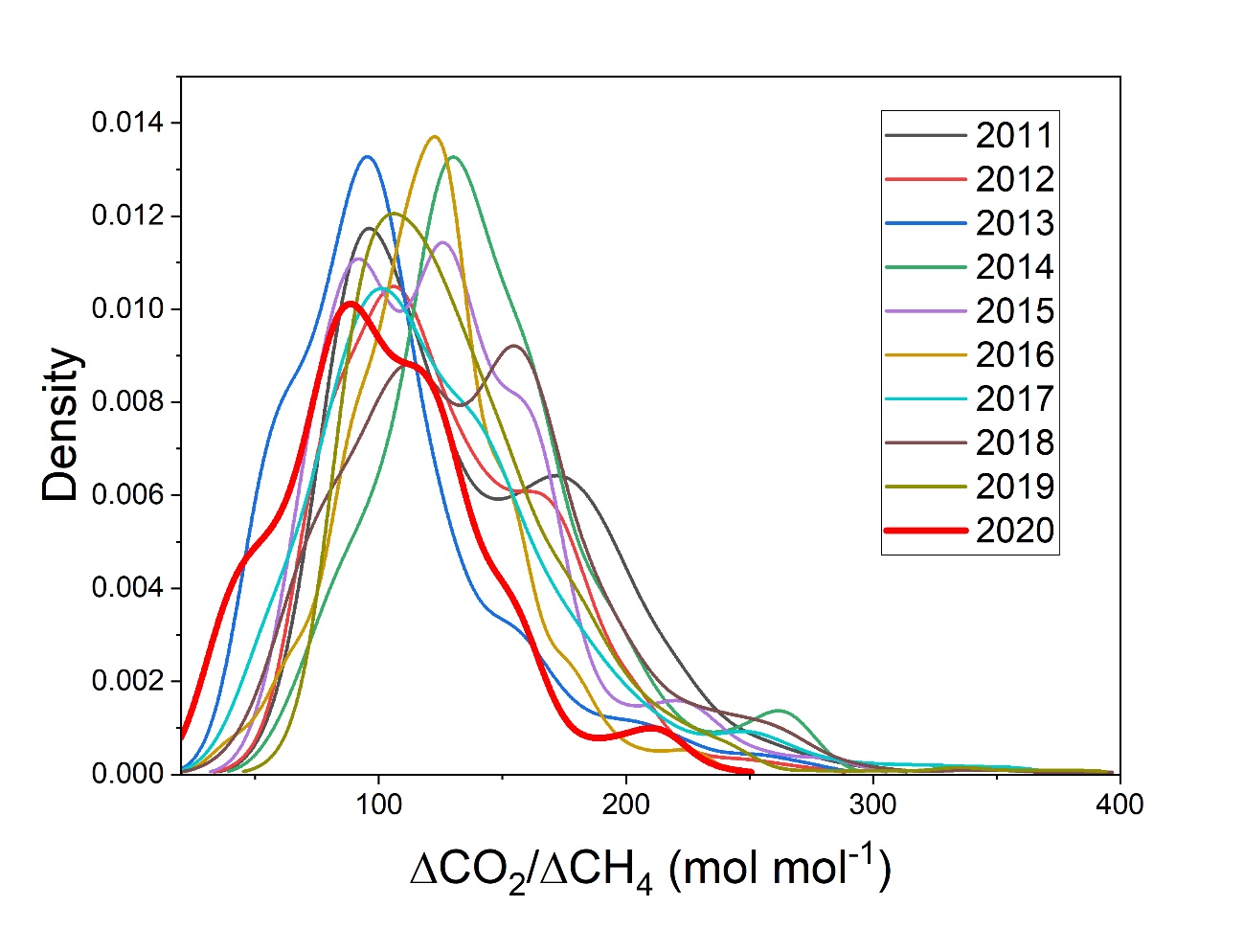


**Fig. S2**. **Histogram of ΔCO_2_/ΔCH_4_ ratios in February of the recent decade.** Each line represents the histogram of the ΔCO_2_/ΔCH_4_ ratios in February for the corresponding year within the recent decade.


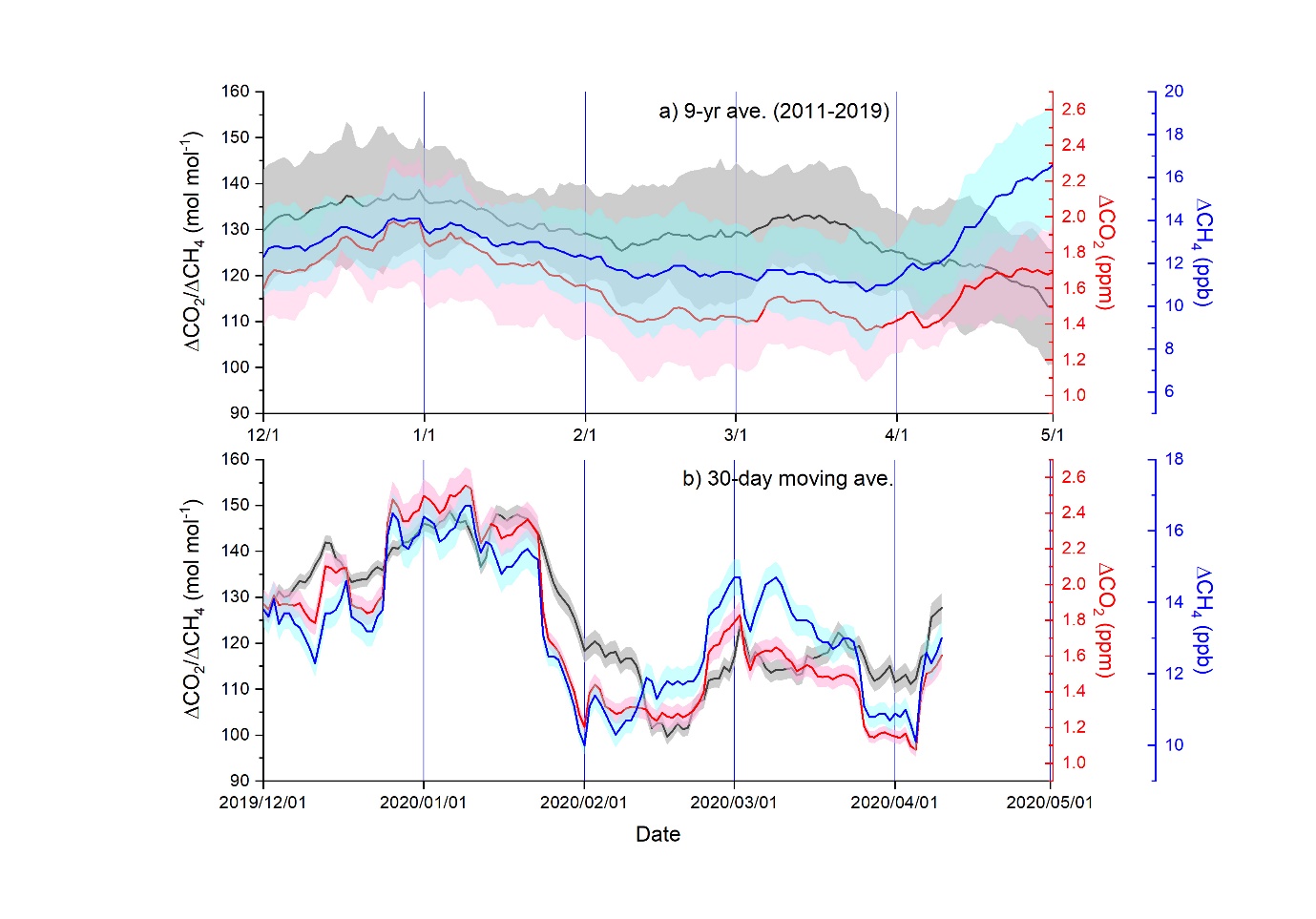


**Fig. S3**. **Temporal variations of CO_2_ and CH_4_ from winter to spring.** (a) 9-year averages of the 30-day moving averages of the ΔCO_2_/ΔCH_4_ ratios (black line), ΔCO_2_ (red line), and ΔCH_4_ (blue line). Grey, pink, and light blue shades represent the range of the corresponding standard deviations (1σ). (b) The 30-day moving average of ΔCO_2_/ΔCH_4_ ratios (black line), ΔCO_2_ (red line), and ΔCH_4_ (blue line) from December 2019 to April 2020. Grey, pink, and light blue shades represent the range of the corresponding standard errors.

**Fig. S4**. **Surface temperature anomalies in eastern China.** Surface air temperature anomalies in East Asia from the NCEP-2 reanalysis for February 2020 (a) and March 2020 (b). (c) Time series of the surface air temperature anomaly over the East China region from where the air mass typically originated during the February-March period for the Hateruma site. The NCEP-DOE Reanalysis 2: Gaussian Grid data are available at: <https://psl.noaa.gov/data/gridded/data.ncep.reanalysis2.gaussian.html>. The maps were generated by using GrADS (<http://cola.gmu.edu/grads/grads.php>).


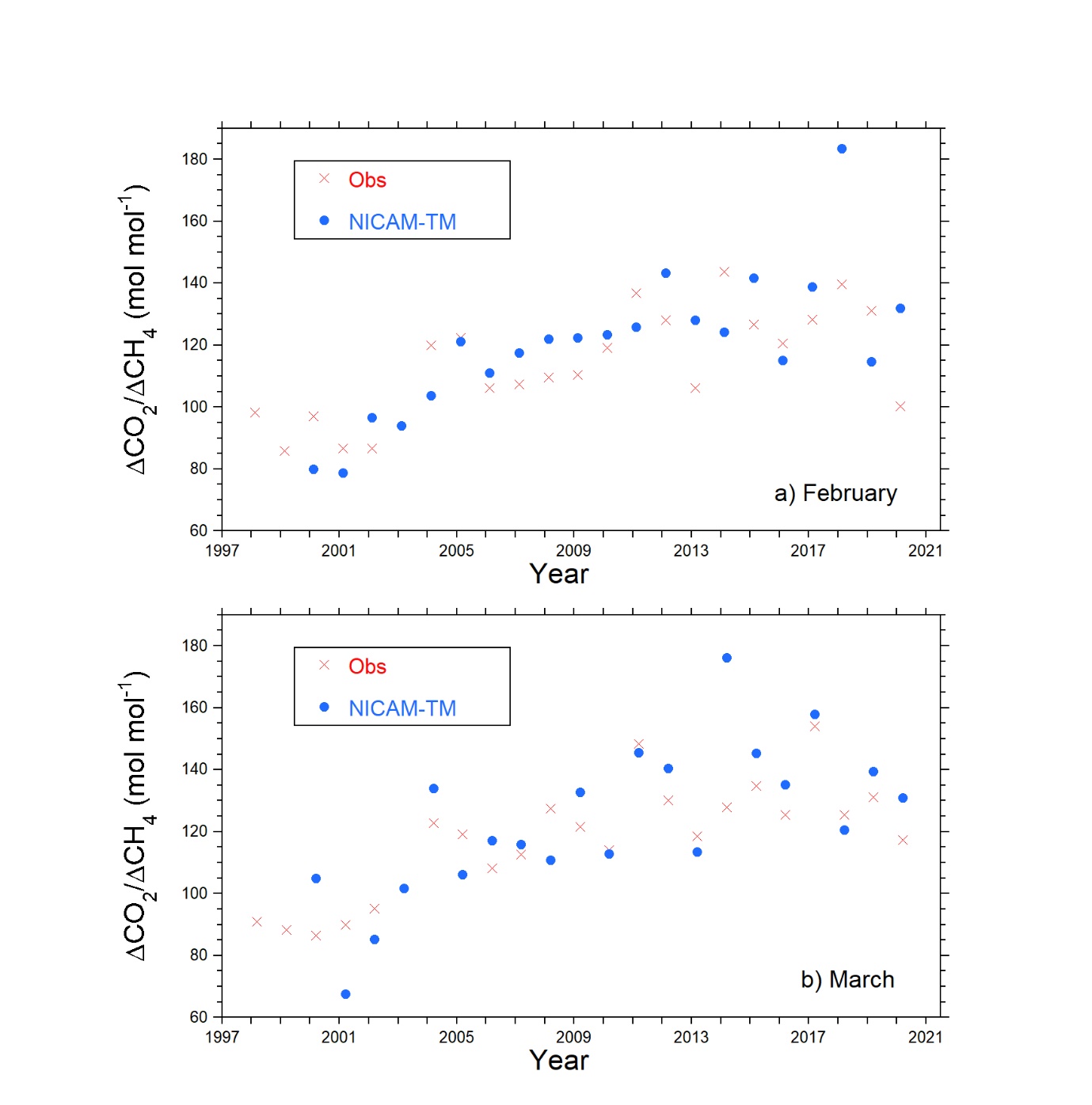


**Fig. S5**. **Comparison between observed and simulated monthly ΔCO_2_/ΔCH_4_ ratios.** Red crosses and blue circles represent the monthly averages of the observed and simulated ΔCO_2_/ΔCH_4_ ratio, respectively, for (a) February and (b) March. In the simulation based on the NICAM-TM, the time-dependent FFCO_2_ emissions were used (the FFCO_2_ in 2018 were repeatedly used for 2019 and 2020), whereas the monthly climatological biospheric CO_2_ fluxes were repeatedly used for the whole period.


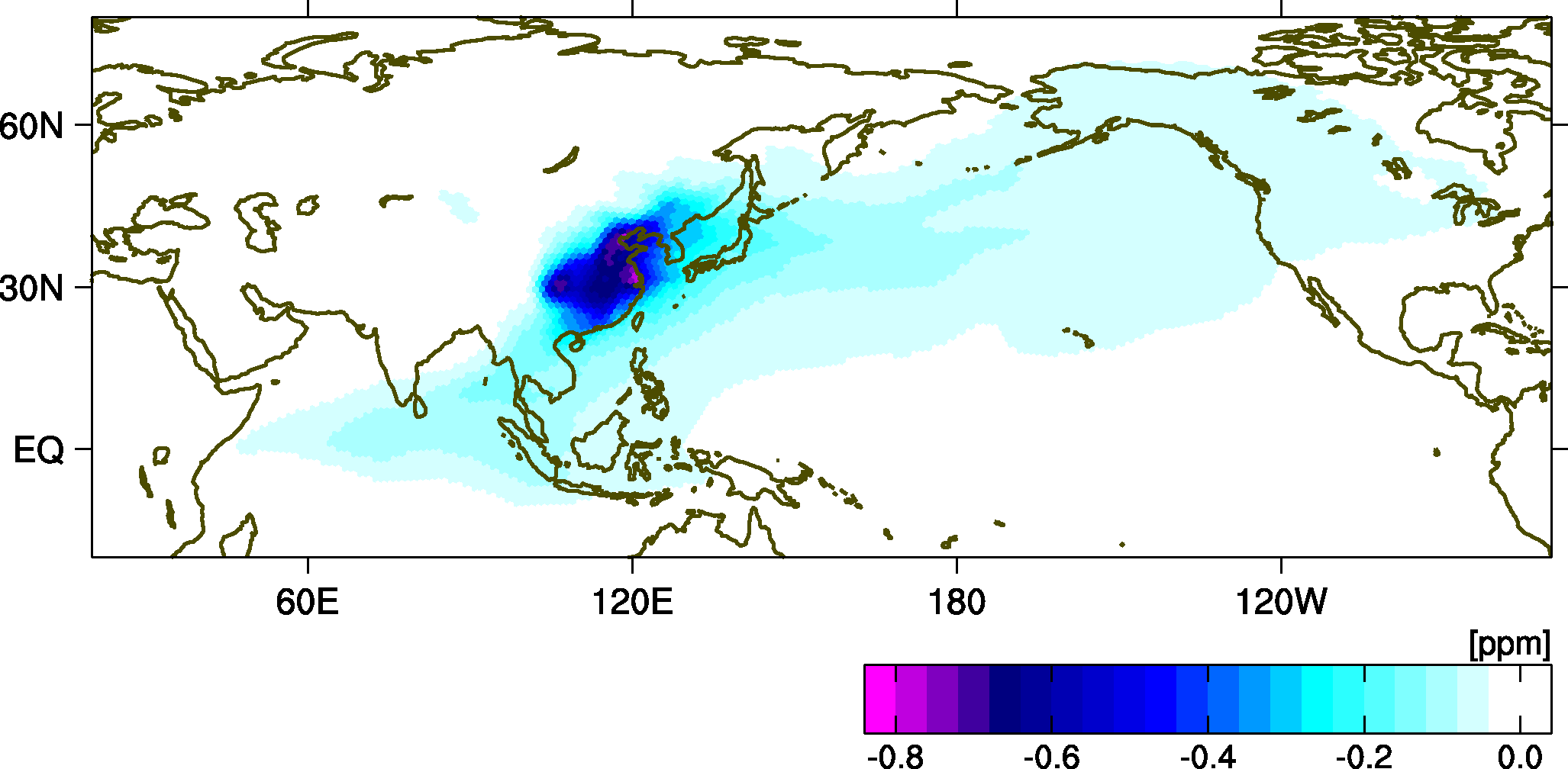


**Fig. S6**. **NICAM-TM-simulated XCO_2_ decrease in February due to COVID-19.** The difference of mean XCO_2_ for February between the control and upper-limit (**-**30% FFCO_2_ emission in February) runs is depicted. The maximum difference is about 0.8 ppm. Note here that the model horizontal resolution is 112 km, which is much larger than the satellite footprints. The figure shows the spread of the emission reduction signal from China to the southwest and northeast directions, indicating how the atmospheric transport mixes regional emissions globally within one month. The map was drawn by using NCAR Graphics libraries ver. 5.1.0 (http://ngwww.ucar.edu/index.html)


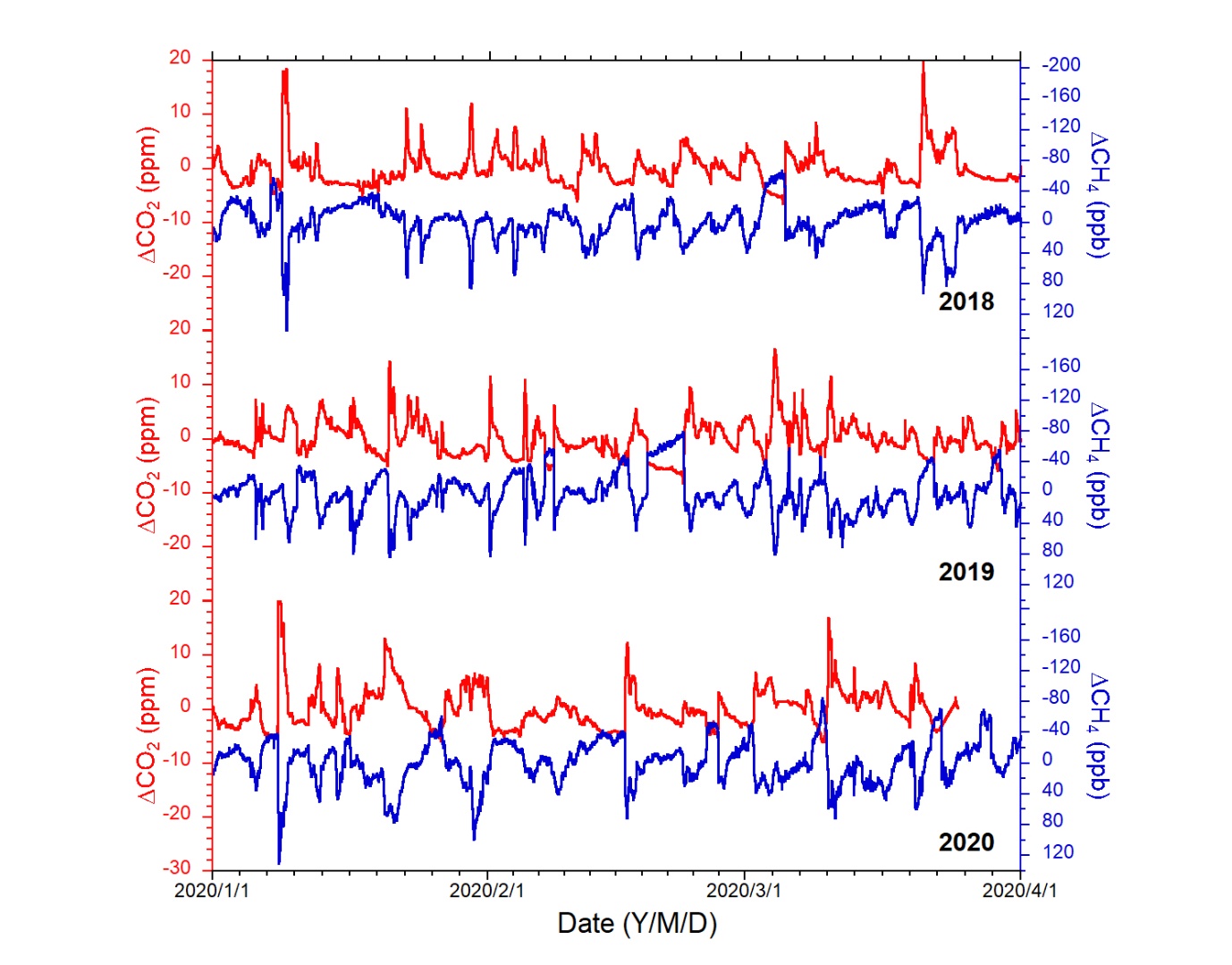


**Fig. S7**. **Temporal variations of the atmospheric CO_2_ and CH_4_ at HAT in winter.** Comparison of detrended and deseasonalized CO_2_ (red lines) and CH_4_ (blue lines) mole fractions from January to March in 2018 (top), 2019 (middle), and 2020 (bottom).


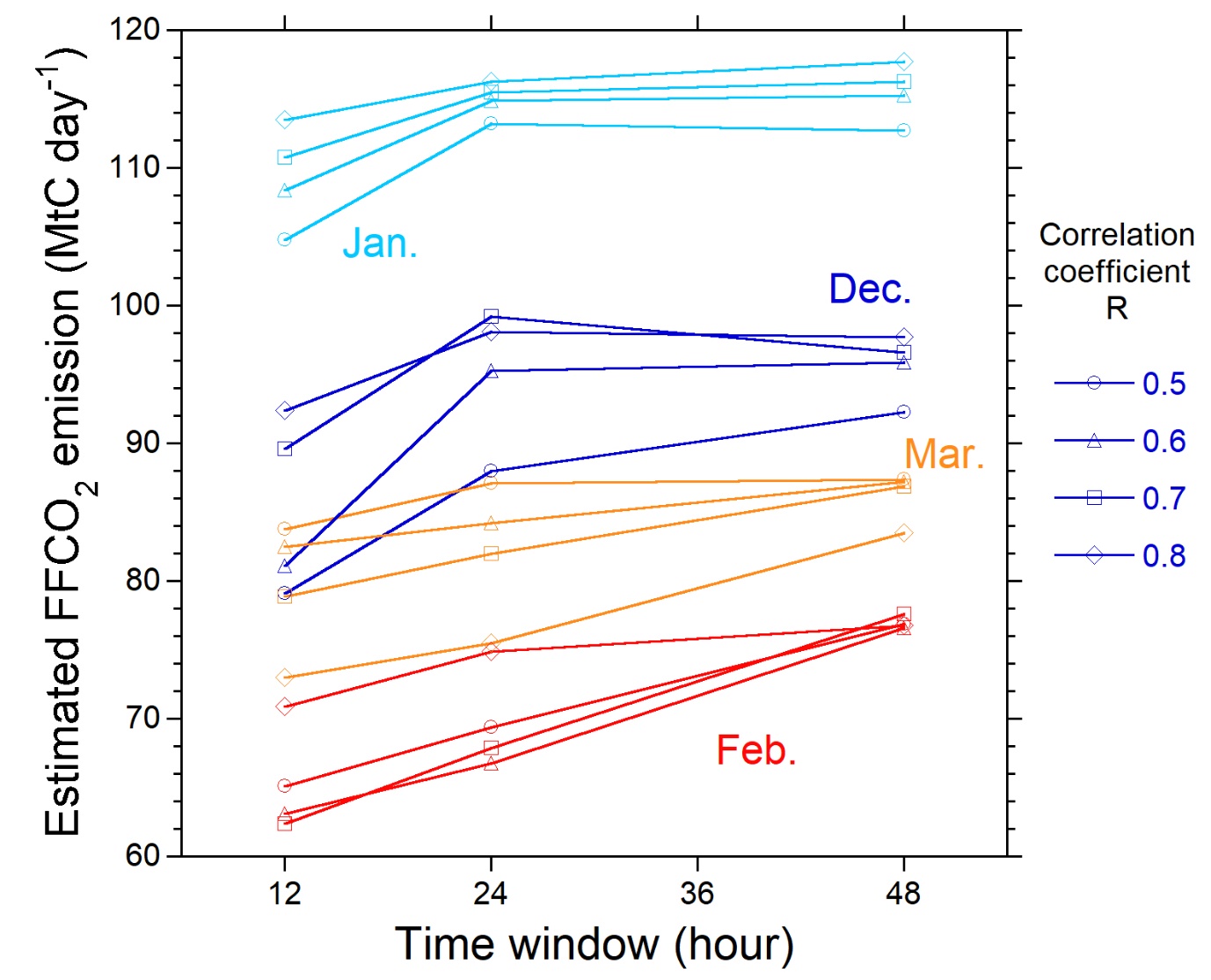


**Fig. S8**. **Dependence of estimated FFCO_2_ emissions on parameters.** The monthly FFCO_2_ emissions from China estimated by the ΔCO_2_/ΔCH_4_ ratios plotted against the duration of the time windows. Each symbol represents a different correlation coefficient used for the data selection.


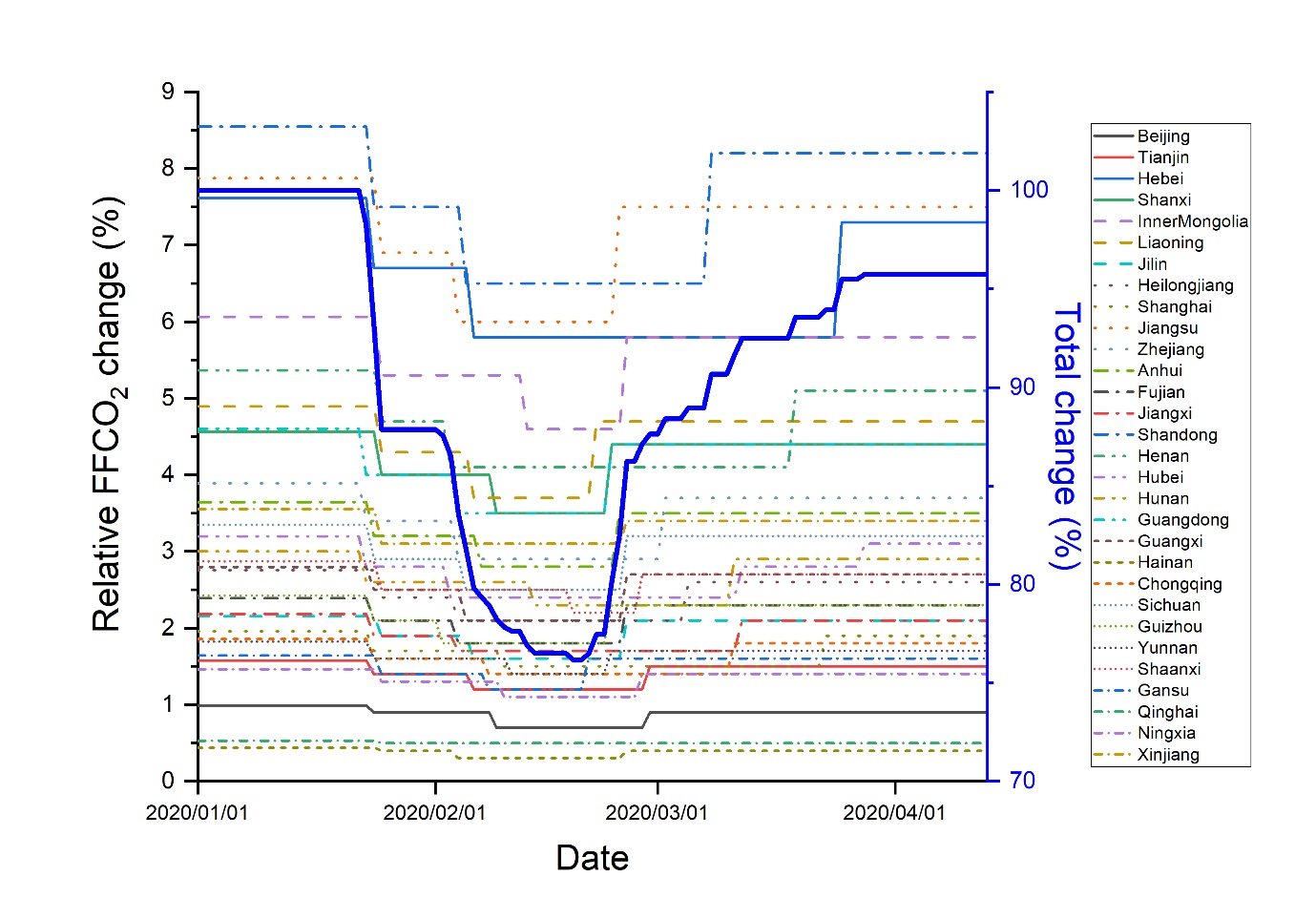


**Fig. S9**. **The changes in the FFCO_2_ emissions from China.** Relative contributions of the FFCO_2_ emissions from 30 Chinese provinces to the total FFCO_2_ emissions based on the time series of the confinement index (CI) reported by Le Quere et al. (2020) (left y-axis) and total FFCO_2_ change (blue thick line, right y-axis).


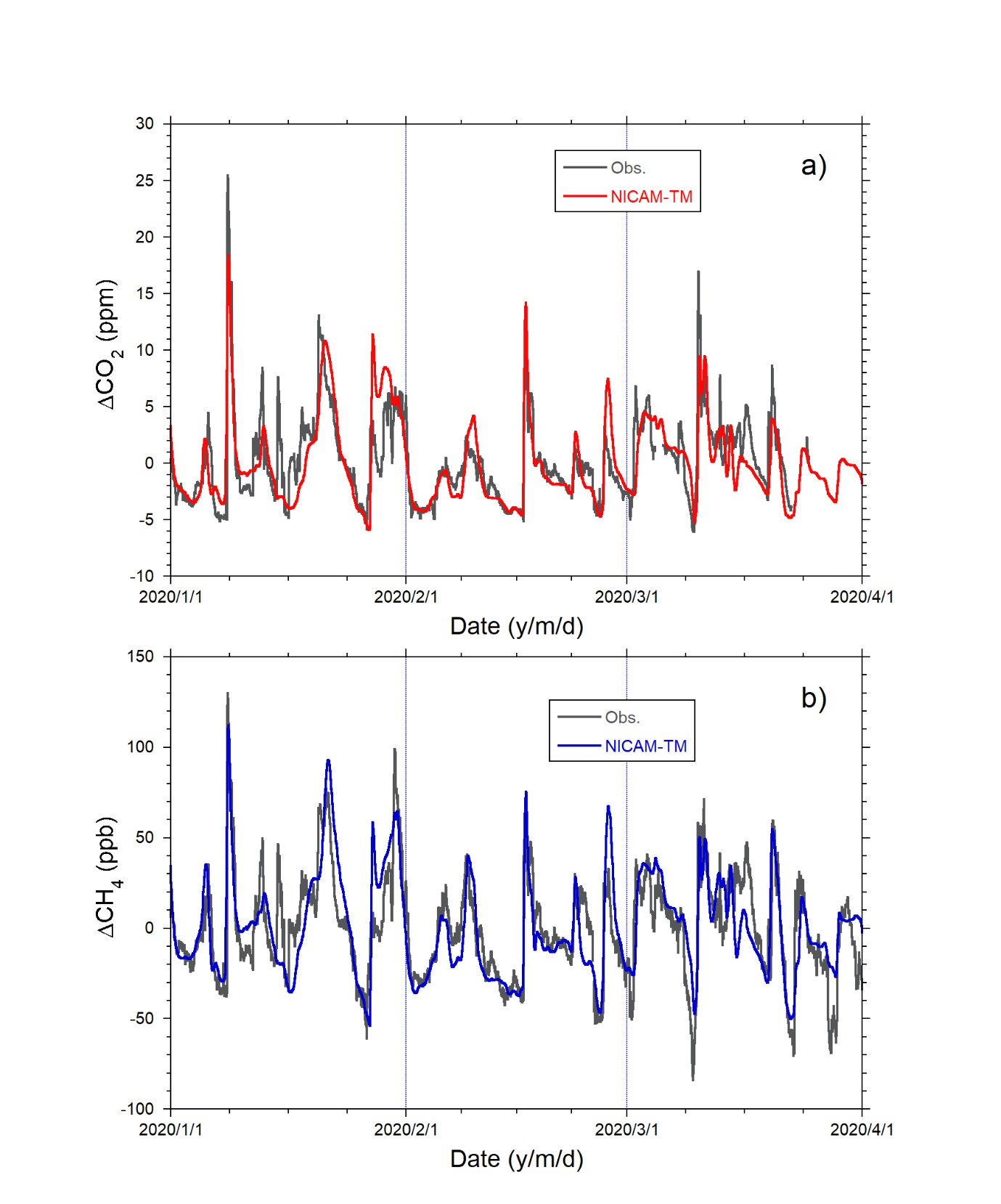


**Fig. S10**. **Comparison between observation and simulation.** Detrended and deseasonalized (a) CO_2_ and (b) CH_4_ mole fractions at HAT from January 2020 to March 2020. Grey lines represent the observed hourly values and red and blue lines represent the hourly CO_2_ and CH_4_ values simulated by the NICAM-TM, respectively.
